# First-principles study of the influence of different interfaces and core types on the properties of CdSe/CdS core-shell nanocrystals

**DOI:** 10.1038/srep10865

**Published:** 2015-06-03

**Authors:** V. Kocevski, J. Rusz, O. Eriksson, D.D. Sarma

**Affiliations:** 1 Department of Physics and Astronomy, Uppsala University, Box 516, S-751 20 Uppsala, Sweden; 2 Solid State and Structural Chemistry Unit, Indian Institute of Science, Bangalore-560 012, India

## Abstract

With the expanding field of nanoengineering and the production of nanocrystals (NCs) with higher quality and tunable size, having reliable theoretical calculations to complement the experimental results is very important. Here we present such a study of CdSe/CdS core-shell NCs using density functional theory, where we focus on dependence of the properties of these NCs on core types and interfaces between the core and the shell, as well as on the core/shell ratio. We show that the density of states and the absorption indices depend rather weakly on the type of interface and core type. We demonstrate that the HOMO wavefunction is mainly localised in the core of the nanocrystal, depending primarily on the core/shell ratio. On the other hand the LUMO wavefunction spreads more into the shell of the nanocrystal, where its confinement in the core is almost the same in each of the studied structural models. Furthermore, we show that the radiative lifetimes decrease with increasing core sizes due to changes in the dipolar overlap integral of the HOMO and LUMO wavefunctions. In addition, the electron-hole Coulomb interaction energies follow a similar pattern as the localisation of the wavefunctions, with the smaller NCs having higher Coulomb interaction energies.

Because of the possibility of producing high quality nanocrystals (NCs) with size tunable band gap, colloidal semiconductor NCs have shown a great potential for applications in single photon sources^1^,^2^, biomedical labelling^3^,^4^, light-emitting diodes (LEDs)^5^,^6^,^7^, and lasers^8^. The as-prepared NCs have a high surface-to-volume ratio, where the surface properties have a significant effect on the photoluminescence (PL) properties of the NCs, especially on the emission efficiency, energy spectrum and time evolution. One way to lower the effect of the surface is to passivate the dangling bonds with organic molecules. Although the NCs capped with organic molecules show high solubility, the failure of the organic molecules to simultaneously passivate both the anionic and cationic surface sites gives rise to mid gap states. Such mid gap states provide rapid, non-radiative decay channels and, as a consequence, lower the quantum efficiency (QE)^9^.

Using a wider band gap semiconductor as capping agent, will ensure full passivation of the dangling bonds on the surface. Moreover, the charge carriers’ tunnelling may be suppressed and excitons can be confined within the core of the NC. The core-shell nanocrystals produced in this way will exhibit high intensity light, with a narrow emission line width, and very high QE. But the lattice mismatch at the interface of the core and the shell NCs can cause some dislocations, which in turn can give rise to non-radiative recombination channels, reducing the QE^10^,^11^,^12^,^13^.

To utilize their PL properties for devices which require bright and constant light (e.g. lasers, tracking applications, single-photon sources), the non-radiative recombination channels need to be reduced as much as possible^1^,^4^,^8^,^14^. To achieve this, one needs to go beyond manipulating the core/shell ratio, and engineer the internal structure of the core-shell structures. This can be done by changing the interface between the core and the shell, e.g. introducing an alloyed^15^,^16^ or a graded interface between them^17^, or by introducing a graded core^18^.

In the past decade a great deal of attention has been focused on the synthesis and experimental analysis of one particular type of core-shell NCs, the CdSe/CdS NCs^15^,^16^,^18^,^19^,^20^,^21^,^22^,^23^,^24^,^25^,^26^,^27^, especially because of their excellent PL properties, emitting high intensity light in a very narrow spectral range. These core-shell structures are built up of CdSe in the core region, whereas the shell region is made from CdS. The CdSe/CdS core-shell NCs have a type I band alignment, where both the hole and the electron are localised in the CdSe core region. However, the electron wavefunction exhibits a higher spread into the CdS shell region, giving rise to a reduced localisation in the CdSe core, compared to the hole wavefunction. This differential localisation of both the electron and the hole gives these NCs a PL with a high QE and high photostability at room temperature. With the expanding number of experimental studies, having a more accurate theoretical model for the core-shell NCs is desirable. This will complement the experimental results, help in better understanding of the fundamental properties of these core-shell structures, and hopefully spark new ideas for structural manipulation.

The CdSe/CdS core-shell NCs have been studied theoretically using effective mass approximation (EMA)^28^, multi-band *k* ⋅ *p* method^29^ and tight-binding (TB) approximation^18^,^30^. Although all these methods are fast and can be used for studying big systems, they are approximate in nature and have difficulties in describing some properties of non-periodic systems, like the NCs^31^,^32^. Therefore performing a reliable detailed first-principles study of CdSe/CdS core-shell NCs is desirable. Here we present a detailed density functional theory (DFT) study of CdSe/CdS core-shell NCs, with different sizes, different interface between the core and the shell, and different core types. We investigate the electronic and optical properties of these NCs, to have an overview of how these properties depend on the different structural models, and to see if this can give information about the PL properties of the NCs. To have a deeper insight into the PL properties, we extend our study by quantifying the localisation of the electron and the hole wavefunctions, and the radiative lifetimes between these states. To complete the study we calculate the electron-hole Coulomb interaction energies for each of the studied structures. It is to be noted that the present work represents the first attempt to theoretically compute these important optical properties of such complex NCs within the first-principles approach.

## Structural models and methodology

**Figure 1:**
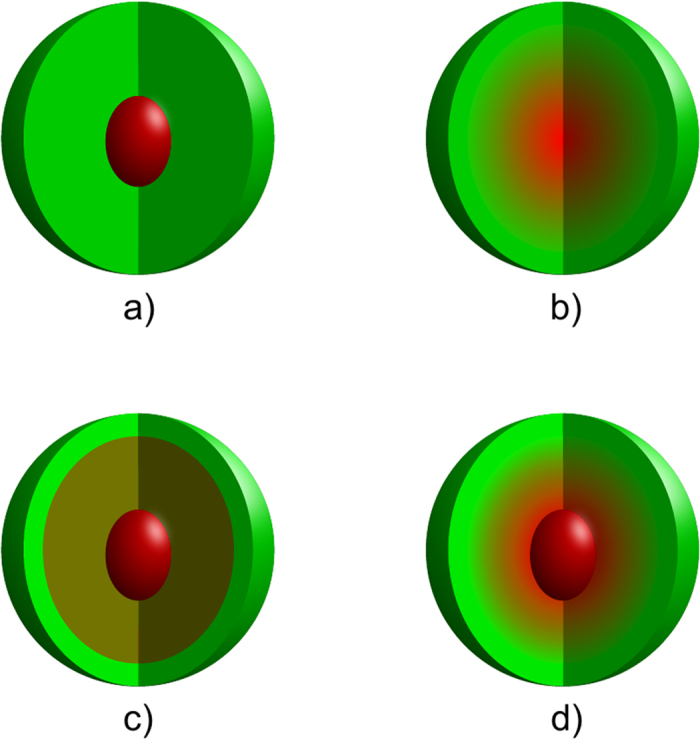
Schematic representation of the four structural models: **a**) pure core; **b**) graded core; **c**) alloyed interface; and d) graded interface.

For the purpose of our study we made CdSe/CdS core-shell NCs with zinc blende crystal structure and a diameter of 3.1 nm, which are large enough to be relevant for comparison to the experimental results^15^. We used four different structural models: pure core, graded core, alloyed interface and graded interface, schematically represented in Fig. 1. Beside the large number of reports of CdSe/CdS core-shell structures with pure core (Fig. 1a) in the literature^16^,^19^,^20^,^21^,^22^,^25^,^27^,^33^,^34^,^35^, the other three structural models, depicted in Fig. 1b–d, have been realised experimentally just recently. For example in Ref. 15 CdSe/CdS NCs with an alloyed interface (Fig. 1c) are experimentally synthesised, and in Ref. 17 structures with graded core (Fig. 1b) and graded interface (Fig. 1d) were reported.

All of these structures have 6 cationic (Cd) and 6 anionic layers (Se, S), with a Cd atom in the center, where the concentration of Se and S atoms in the anionic layers are adapted to the given structural model. The structure with a graded core is made by changing the Se concentration in all of the anionic layers from 1, in the center, to 0, in the surface shell, with a step of 0.2. The resulting graded core, excluding the surface shell, has a diameter of 2.2 nm. The structures with an alloyed and graded interface have a core consisting of 2 Cd and 2 Se layers, with a diameter of 0.8 nm, and an interface made of 3 Cd and 3 anionic layers. In the case of an alloyed interface, the Se atoms in the interface are randomly substituted with S atoms. To have a similar number of Se and S atom in each of the structures, the concentration of the S atoms in the alloyed interface is made to be 0.6 (see Table 1 for more details). In the case of the graded interface the Se concentration changes from 0.75, in the layer closest to the core, via 0.5 and 0.25 in the layer next to the surface shell. In both structures, the size of the core and the interface region together is 2.2 nm.

**Figure 2:**
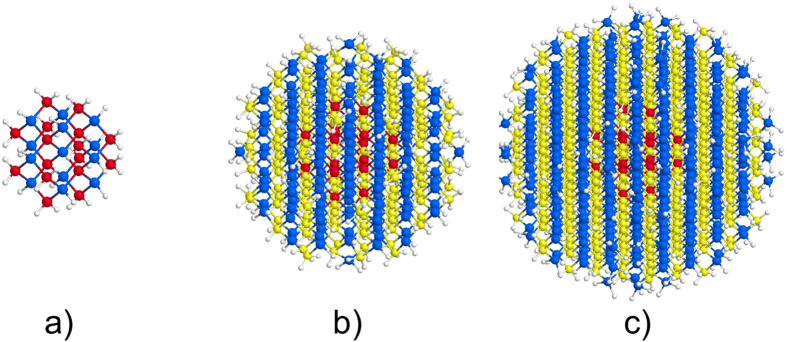
Ball models of relaxed: **a**) 1.1 nm CdSe NC; **b**) 2.4 nm CdSe/CdS core-shell NC with pure core; and **c**) 3.1 nm CdSe/CdS core-shell NC with pure core. The Cd, Se, S and the pseudohydrogen are shown in blue, red, yellow and light-blue colours, respectively.

To have a better understanding how the properties of the NCs are affected by the core-shell ratio and for comparison with the other structural models, we made structures with three different diameters of the pure core: 0.8 nm, 1.55 nm and 2.2 nm. To have an overview how the properties are changing with the size of the NCs, we also made two smaller structures, where the entire NCs have a diameter of 2.4 nm. Following the experimental study of CdSe/CdS NCs with alloyed interface^15^, one of the structures was made by removing the capping CdS shell from the structure with alloyed interface, leaving only a 0.8 nm CdSe core and an alloyed shell. The other structure was made by putting a CdS shell as thick as the alloyed shell on top of 0.8 nm CdSe core, making a core-shell NC with pure core. As a reference we used a pure CdSe core as a NC. Shown in Fig. 2 are models of the pure CdSe NC, and CdSe/CdS NCs with pure core and different sizes.

The calculations were performed using the pseudopotential DFT package SIESTA^36^,^37^, which uses numerical atomic orbitals as the basis set, allowing for very efficient, but still reliable calculations. We used the local density approximation (LDA) exchange correlation potential, with Ceperley-Alder parametrisation. The basis set for each of the atomic species is double-*ζ*, including one set of polarization orbitals (DZP). For each of the structures, the atoms on the surface that have only one neighbour were removed, and the surface was capped with pseudohydrogen, to ensure full passivation of the dangling bonds. This way of surface passivation give us the opportunity to focus only on the changes in the properties arising from the different structural models, neglecting the effects originating from surface imperfections or the capping layer. The atom positions were relaxed until all forces on the atoms were lower than 0.04 eV/Å. The volume of each structure is calculated as the volume of a convex hull drawn around the structure. The diameter of the NC is calculated as a diameter of a sphere that has the same volume as the NCs’ structure. The diameters of the cores are calculated in a similar fashion as the diameter of the NCs. In Table 1 the diameters of relaxed NCs and the cores, including the number of Cd, Se and S atom in the NCs, are summarised.

## Results and discussion

### Electronic and optical properties

**Figure 3:**
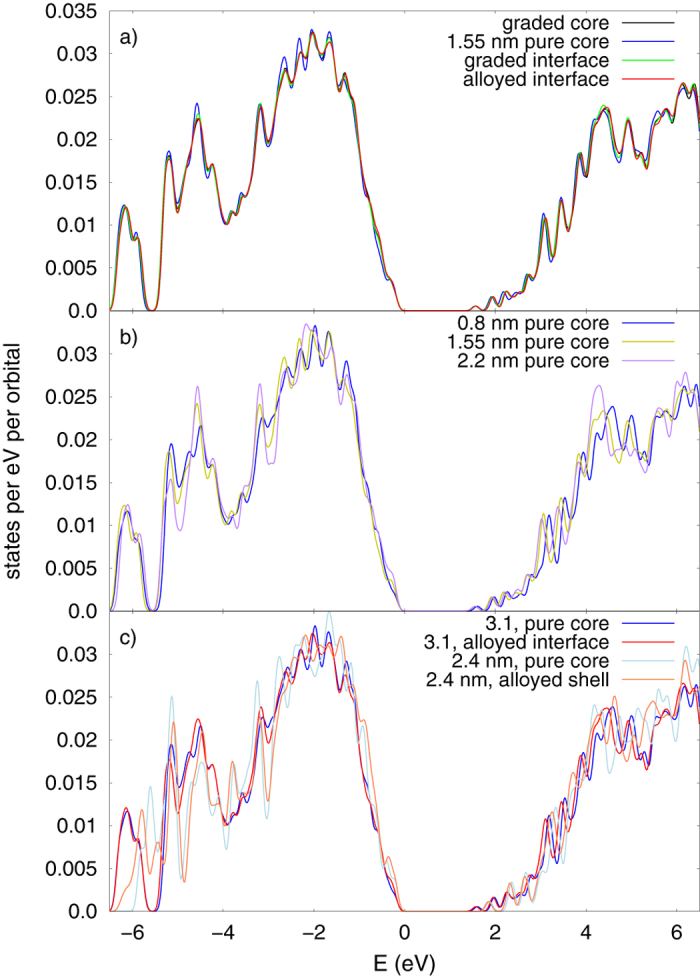
Density of states of: **a**) 3.1 nm NCs, made with different structural models; **b**) 3.1 nm NCs with different sizes of the pure core; and **c**) 3.1 nm and 2.4 nm NCs.

For initial characterisation of the electronic structure of the CdSe/CdS NCs, we have evaluated the density of states (DOS) of the relaxed structures, and their dependence on the structural model, the size of the core and the size of the NCs. In Fig. 3 we show the DOS of 3.1 nm and 2.4 nm NCs, as a function of the structural model, and the size of the core.

Comparing first the DOS of 3.1 nm NCs with different structural models, Fig. 3a, it is noticeable that there is surprisingly little of difference between their DOS. Although the different layers in each of the structures have different number of Se and S atoms, the total number of Se and S atoms in the structures is almost the same (see Table 1). Having in mind that the DOS represents the electronic structure of the material as a whole, small changes in the number of Se and S atom will have a little effect on the DOS.

Having this in mind, we compare the DOS for NCs with same size, but different core sizes. Such structures differ more significantly in the count of Se and S atoms, respectively. Yet, it is evident from Fig. 3b that the difference in the DOS remains rather small. Although by changing the size of the cores the number of Se and S atoms is changing, the contribution to the density of states from the Se and S atoms is similar (see Fig. 8 below), which in turn causes only minor changes in the total DOS.

Finally, we investigate how the DOS depend on the size of the NCs. We compare the DOS of 3.1 and 2.4 nm NCs, both having pure core and alloyed interface, in Fig. 3c. One can see that the dominant features of the DOS remain very similar, except for the valence band of the 2.4 nm NCs, which appears somewhat compressed, compared to the one of the 3.1 nm NCs. In addition, there is a widening of the band gap in the smaller NCs. Both observations are a direct consequences of the well-known quantum confinement^38^ effect, according to which the band width becomes smaller and the band gap gets bigger^39^ with a decreasing size of the NCs.

**Figure 4:**
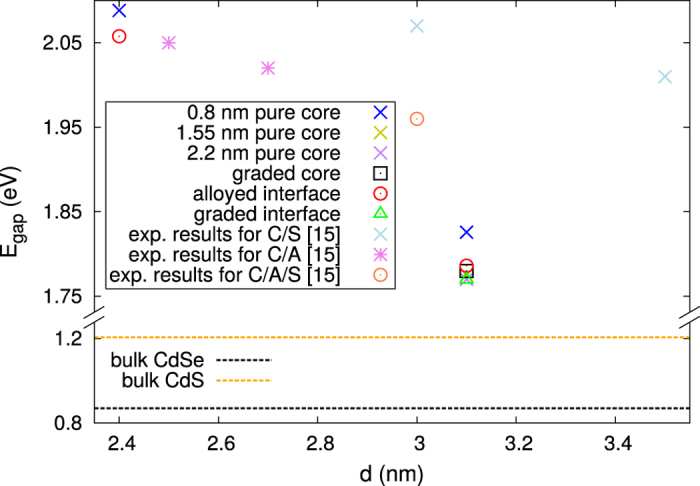
Band gaps of CdSe/CdS core-shell NCs with different size, made using different structural models and core sizes. The 1.1 nm pure CdSe NC has 3.6 eV band gap (not shown on the figure). The experimental gaps, taken from Ref. 15, correspond to core/shell (C/S), core/alloyed shell (C/A), and core/alloyed interface/shell (C/A/S) structures, all of which have 1.5 nm CdSe core. The band gaps for bulk CdSe and CdS are shown with dashed black and orange lines, respectively.

For a closer look on the effects of quantum confinement and the structural model on the band gap, we extracted the band gaps from the DOS as the energy difference between the lowest unoccupied and the highest occupied eigenstate in the NCs. In Fig. 4 the band gaps are presented as a function of the NCs’ diameter, together with band gaps taken from experiments^15^. The most noticeable observation is the increase of the band gap, as the size of the NCs is reduced from 3.1 nm to 2.4 nm, as a consequence of the quantum confinement. Dependence of the band gap on the particular core-shell interface is minor, as could be expected from Fig. 3a,b. Comparing the band gaps of NCs with the same size, it is evident that the band gap of the NCs with 0.8 nm core is larger than the band gap of the rest of the NCs. This difference in the band gaps can be attributed to the lowering of the concentration of the smaller band gap material, CdSe, in the NCs with 0.8 nm core. Similarly to the experimental gaps, the band gap of the 3.1 nm NC with sharp interface is bigger than the band gap of the NC with alloyed interface. However, it should be noted that the theoretical band gaps are lower than the experimental ones, especially for bulk CdSe and CdS, which is a known underestimation of the band gaps for the used LDA functional^40^,^41^.

**Figure 5:**
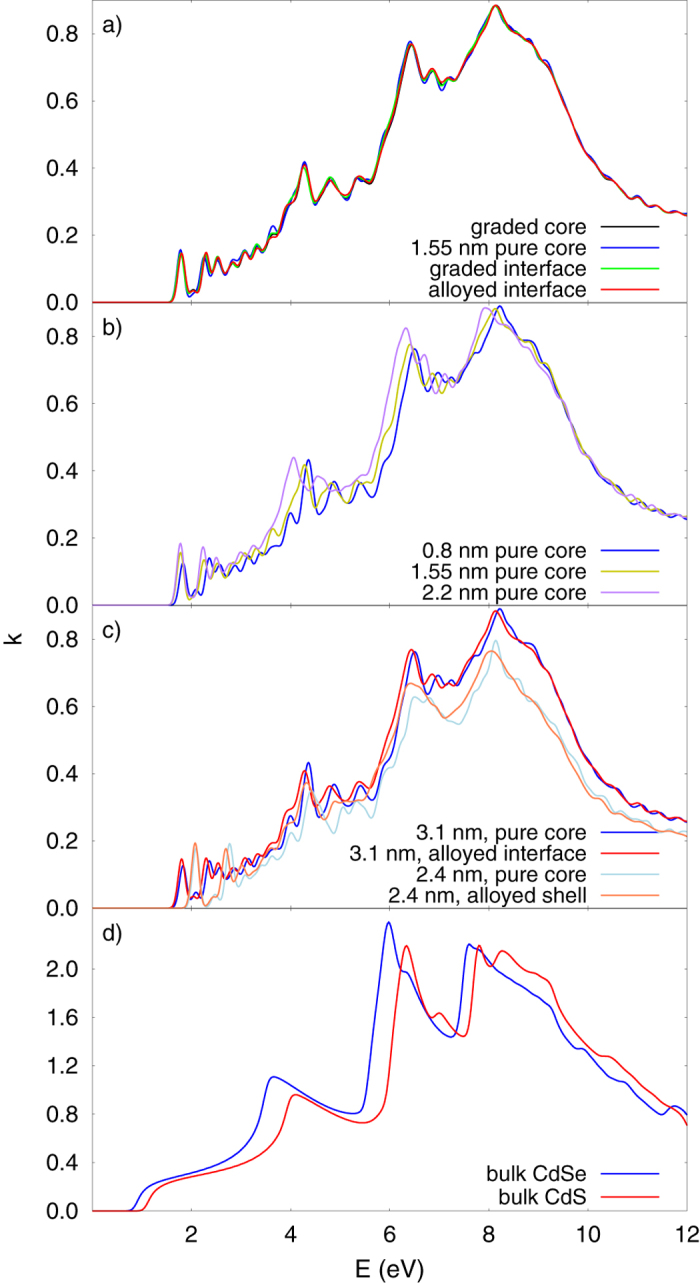
Absorption indices of: **a**) 3.1 nm NCs, made with different structural models; **b**) 3.1 nm NCs with different sizes of the pure core; **c**) 3.1 nm and 2.4 nm NCs with pure core and alloyed interface; and d) bulk CdSe and CdS.

In addition to the study of the electronic structure, we calculated the imaginary part of the dielectric tensor, to get some more insight into the optical properties of these NCs. The imaginary part of the dielectric tensor was obtained by calculating the dipolar transition matrix elements between unoccupied and occupied single-electron eigenstates, within an energy interval of 13 eV, as implemented in SIESTA^36^,^37^. A Gaussian broadening of 0.1 eV was used when doing the calculations. The real part of the dielectric tensor was obtained using the Kramers-Krönig transformation, after which it is possible to calculate the absorption indices—shown in Fig. 5. Similar to the DOS, the absorption indices of the structures with different interfaces (Fig. 5a) are very similar to each other, and do not differ much from that of bulk CdSe or CdS (Fig. 5d). However the absorption indices of the NCs with different core size are slightly different, showing a higher absorption as the size of the core gets bigger. This change comes from the larger absorption of CdSe compared to that of CdS, see Fig. 5d. Comparing the NCs with different sizes the most obvious differences is in the onset of the optical absorption, which shifts to higher energies for decreasing size of the NCs. A decrease in the absorption with an increase of the NCs size is also noticeable. Both of these observations are consequences of the quantum confinement effect.

We see that neither the electronic nor the studied optical properties show a big sensitivity on the structural model or the size of the NCs. Furthermore, we are interested in the study of the experimentally observed PL peak, particularly the transition between the lowest unoccupied (LUMO) to the highest occupied (HOMO) eigenstate, i.e. single-exciton recombination. Because the recombination of the single-exciton is dependent on the overlap between the LUMO and HOMO wavefunctions (WFs), we also investigate the spatial distribution of the LUMO and HOMO WFs, and the radiative lifetimes. Including higher order excitonic processes can give a further insight into the PL properties of these NCs. However, calculations like that are very demanding, and with the current computational capabilities almost unachievable for large systems, as the ones modelled in our study.

### Spatial distribution of the wavefunctions

**Figure 6:**
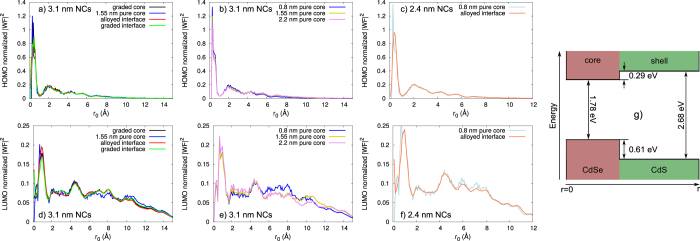
Spatial distribution of the normalised HOMO and LUMO WFs density and band alignment in CdSe/CdS core-shell NCs. **a**–**c**) radial distribution of the normalised density of HOMO WF; **d**–**f**) radial distribution of the normalised density of LUMO WF; g) schematic representation of the band alignment in CdSe/CdS core shell NCs. The offsets are calculated from the separate projected density of states of the core and the shell, of 3.1 nm NC with 1.55 nm core, by taking the energy at which the PDOS around the gap are higher than 0.002 states per eV per orbital.

As mentioned before, the CdSe/CdS core-shell NCs have a type I band alignment, which should cause the electron and hole WFs to be confined dominantly within the core of the NC. This higher confinement of the WFs within the core of the NCs causes a reduction of the surface effects, more specifically a reduction in the influence of the non-radiative recombination channels and in the probability of photo-oxidation of the hole, giving rise to a higher QE. Therefore, it is very important to know the extent of the confinement of the HOMO and LUMO WFs within the core of the NC, compared to the whole NC. To find this out, we first calculated the radial distribution of the normalised HOMO and LUMO WF densities, and plotted them as a function of the structural model and size of the NCs—see Fig. 6a–f. The most noticeable is the difference in the distribution of the HOMO and LUMO WFs, where the HOMO WF is localised in the core of the NC, whereas the LUMO WF shows a much wider distribution over the whole NC volume.

Comparing the distribution of the HOMO and LUMO WFs in the 3.1 nm NCs with different core sizes, Fig. 6a,b,d,e, it is evident that both WFs exhibit small changes depending on the core size. As the core gets bigger, the HOMO WF spreads more towards the shell of the NC, lowering the WF density in the core of the NC. On the other hand, the LUMO WF shows a slightly different behaviour, with the WF density in the core and the shell being affected by the core size. This difference in the distribution of the HOMO and LUMO WFs, with an increasing core size, can be attributed to the different confinement potentials of the HOMO and LUMO WFs, see Fig. 6g. The confinement potential of the HOMO WF is much bigger compared to the one of the LUMO WF, confining the HOMO WF in the core of the NCs, and thus giving the possibility of higher delocalisation within the core, with increasing core size. The LUMO WF confinement potential is much smaller, which leads to a higher delocalisation of this WF in the shell, as the shell size grows, slightly lowering the WF density in the core. In the NCs with different structural models, the distribution of the HOMO and LUMO WFs are very similar to each other, which is a consequence of the similar number of Se and S atoms in the structures.

**Figure 7:**
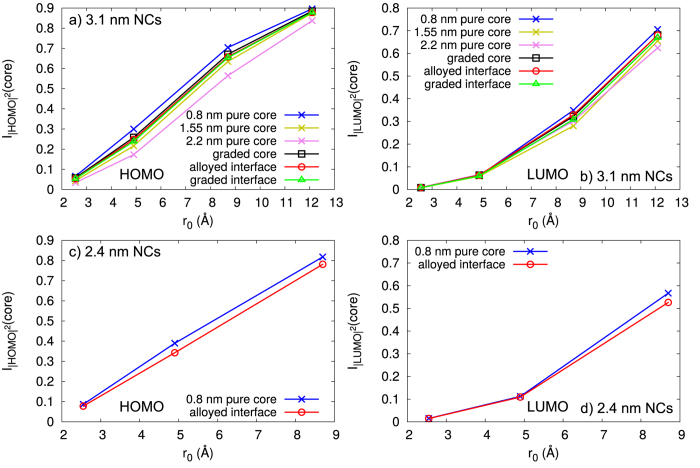
Comparison between the confinement of the HOMO and LUMO WFs, of the 2.4 and 3.1 nm NCs, as a function of the core integration radius, r0: **a**) HOMO WF of 3.1 nm NCs; **b**) LUMO WF of 3.1 nm NCs; **c**) HOMO WF of 2.4 nm NCs; and **d**) LUMO WF of 2.4 nm NCs.

Although this shows some qualitative trends of the changes of the confinement of the HOMO and LUMO WF, it does not give any quantitative information about the extend of confinement in each core. Therefore, we calculate the confinement of the HOMO and LUMO WFs as 
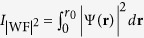
. Note that the integration is performed over the square of the WF itself, Ψ(***r***), and not the WF density as was shown in Fig. 6. To eliminate the effect of using different core sizes, and to see how much of the WFs are confined within the same volume, regardless of the structural model, we have assumed the same core integration radius (*r*_0_) when doing the calculations. We chose four different values for the *r*_0_, 2.6 Å, 4.9 Å, 8.7 Å, and 12.1 Å, which correspond to the distance from the center to the most distant Se atom in CdSe_4_ tetrahedra, and in the smallest, middle sized and biggest pure core, respectively. The resulting dependence of the confinement of the WFs on the *r*_0_ is shown in Fig. 7.

Note that the order of the confinement of the HOMO WF at a given *r*_0_, in the different structures, is kept constant with growing *r*_0_. Keeping in mind the discussion above about the confinement of the WFs and the QE, we would expect that the QE will be higher in structures with smaller core. Also, in the structures with same size, where the core to shell ratio is smaller, a higher QE is expected. Following what was said before, we would expect smaller NCs to have a higher QE. Indeed Bae *at al.*^15^ have shown that as the shell is getting thicker, in the NCs with same core size, the QE of the NCs is getting smaller.

Comparing the confinement of the HOMO WF within the structure with graded core and the one with a pure core of the same size, it is noticeable that there is higher confinement of this WF in the structure with graded core, in the considered *r*_0_ region. This was previously shown in a tight-binding study^18^, now we confirm this result on the basis of parameter-free DFT calculations. Moreover, the difference between the confinement of the HOMO WF in the structures with similar number of Se and S atoms (see Table 1), but different core and interface types, appears rather small. From the small difference in the confinement, one would expect that these structures would have similar QE. This also has been observed experimentally by Bae *et al.*^15^, showing that having a pure core (referred as structure with sharp interface in the paper) or an alloyed interface has rather weak influence on the single-exciton properties of the NCs.

**Figure 8:**
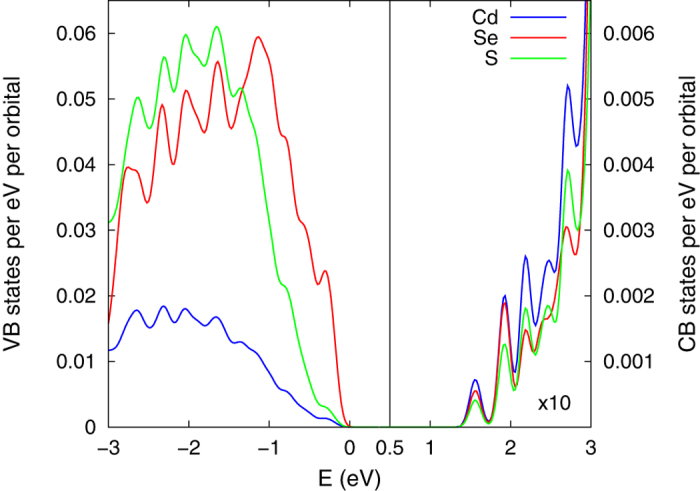
Projected density of states (PDOS) per atom type of 3.1 nm CdSe/CdS core-shell NC with 1.55 nm core. The states of Cd, Se and S atom species are shown in blue, red and green, respectively. The conduction band (CB) states are multiplied by 10 to more clearly distinguish between the different atomic states.

However, when comparing the confinement of the HOMO WF in the 3.1 nm NCs with same core size, 0.8 nm, and sharp and alloyed interface, it is evident that the NC with the alloyed interface has a smaller confinement. A similar behaviour of the confinement of the HOMO WF is noticeable for the NC with the graded interface, where the confinement is practically the same as in the NC with alloyed interface. This difference in the confinement can be understood by looking into the contribution from the different atom types to the HOMO state. Considering that the contribution to the HOMO state comes mainly from the anionic orbitals (see Fig. 8), the change in the number of different anions in the interface region will ultimately cause changes in the spread of the HOMO WFs. Hence, the introduction of Se atoms in the interface region, i.e. making an alloyed or a graded interface, will soften the confinement potential of the HOMO WF, allowing increased spread of the HOMO WF, compared to the NC with the same core size and sharp interface. According to the EMA the Auger lifetimes are inversely proportional to the square of the Fourier component of the HOMO WF calculated at spatial frequency of the final state^42^. Increased confinement of the HOMO WF leads to more spread Fourier components and thus can reduce the Auger lifetimes. With this in mind, based on our calculations of the HOMO WF confinement, we expect that the NCs with alloyed and graded interfaces will have longer Auger lifetimes, compared to the NCs with same size and same core size. This was experimentally observed by Bae *et al.*^15^, showing that Auger channels are suppressed by the interfacial alloying, which was assigned to the smoother confinement potential in the structures with alloyed interfaces.

Furthermore, in their study, Bae *et al.*^15^ have also shown that the structure with a alloyed shell has significantly lower QY, compared to the structure with same size and CdS shell. They attribute this difference to the rising interaction between surface traps and core-localised holes in the NC with alloyed shell. Unlike the experiment, the surface in our models is fully passivated, ensuring that there are no dangling bonds, and moreover, the models have almost perfect structure, with no defects, effectively removing the surface traps from our consideration. This difference in the experimental and theoretical results reassures that the surface indeed plays an important role in the PL properties of the NCs, and having crystalline shell, with as good as possible surface coverage, will yield NCs with higher QE. In addition, it also shows how important it is to confine the HOMO and LUMO WFs within the core, as far from the surface as possible.

On the other hand, the confinement of the LUMO WF in all of the structures, with same size, is very similar, regardless of the used *r*_0_. It is worth noticing that the values for the confinement of the LUMO WF are much smaller compared to the one of the HOMO WF, when the same *r*_0_ is used, which follows our assessment of higher delocalisation of the LUMO WF in the whole NC. This has been termed^18^ a differential collapse of the HOMO WF compared to the LUMO one in the literature. The difference between the confinement of the HOMO and the LUMO WFs is a consequence of the materials used to make the core-shell NC. In the CdSe/CdS core-shell NCs, with same size, the number of cations (Cd) is kept constant, while the number of different anions, Se or S, is changing. As was mentioned before, the HOMO WF is largely influenced by the change in the number of different anions, which will also induce changes in the spread of the WFs. On the other hand, the contribution to the LUMO state comes from both the cationic and anionic orbitals (see Fig. 8). Although the anionic orbitals contribute to the LUMO WF, and the change in the number of Se and S atoms are expected to yield change in the LUMO WF, the contribution from both Se and S are very similar. This similarity in the contribution of the anionic orbitals to LUMO WF will eventually make the spread of the LUMO WF confinement very similar, in all structural models with the same diameter.

### Radiative lifetimes

**Figure 9:**
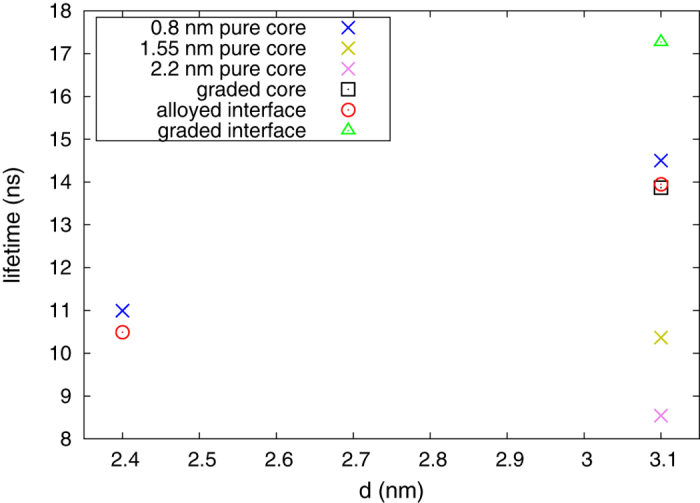
Radiative lifetimes of CdSe/CdS core-shell NCs with different size, made using different structural models and core size.

The difference in the spatial distribution of the HOMO and LUMO WF will influence their dipolar overlap integral, *θ*_*eh*_, and ultimately cause changes in the radiative lifetimes. Having in mind that the radiative lifetimes depend inversely on *θ*_*eh*_ 43, it is expected that the structures with similar spatial distribution of both the HOMO and LUMO WF have similar radiative lifetimes. To see if this is the case, we evaluated the dielectric matrix elements and used them to calculate the radiative lifetimes. Optical emission of the CdSe/CdS core-shell NCs is dominated by transitions between the top of the valence band and the bottom of the conduction band, therefore for the purpose of our study we only calculated the dielectric matrix elements between the HOMO and the LUMO states. The transition rates, 1/*τ*, were calculated using the equation derived from the Fermi’s golden rule^44^:


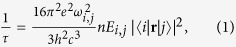


where *E*_*i*,*j*_ is the energy difference between the initial, 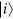, and final, 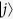, state of the electron, *ω*_*i*,*j*_ is the emission frequency, *h* is the Planck constant, and *c* is the speed of light in vacuum. The refractive index of the NC, *n*, is estimated from the calculated dielectric tensor. From the transition rates we evaluated the radiative lifetimes, and the calculated lifetimes are shown in Fig. 9. Interestingly, the calculated radiative lifetimes are in the range of 8-18 ns, which is in excellent agreement with the experimentally reported values for similar systems^15^,^16^,^17^,^18^.

Evidently, the growing core size, in the 3.1 nm NCs, induces shortening of the radiative lifetimes, which can be attributed to the growing HOMO-LUMO dipolar overlap integral. As we discussed previously, with an increasing core size, the HOMO WF spreads more in the shell, whereas the distribution of the LUMO WF remains relatively unchanged with changes in the core size. This increased spread of the HOMO WF and slight confinement of the LUMO WF enhances the HOMO-LUMO dipolar overlap integral, and consequently lowers the radiative lifetimes. In addition, with a growing NC size (e.g. from 2.4 nm to 3.1 nm in Fig. 9), the radiative lifetimes increase, which is a consequence of the decreased overlap of the HOMO and LUMO WFs with increasing NC size^45^. On the other hand, the structures with different structural models, and similar number of Se and S atoms, have comparable radiative lifetimes. This can be also explained using the reasoning above that the confinement of the HOMO and the LUMO WFs is almost the same in these structures, inducing similar dipolar overlap integrals, which gives similar radiative lifetimes. This observation is in agreement with the experimental results^15^, where it is shown that NCs with sharp and alloyed interface have similar radiative lifetimes.

### Electron-hole Coulomb interaction energies

A more non-local characteristic of the excitation is given in terms of the electron-hole (e-h) Coulomb interaction energies (*E*_*b*_). In general, the Coulomb interaction energy characterizes the energy of attraction between the electron (LUMO) and the hole (HOMO). We evaluated the Coulomb interaction energies using the equation given by Wang *et al.*^46^:


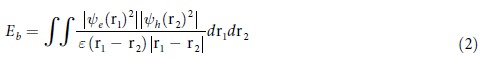


**Figure 10:**
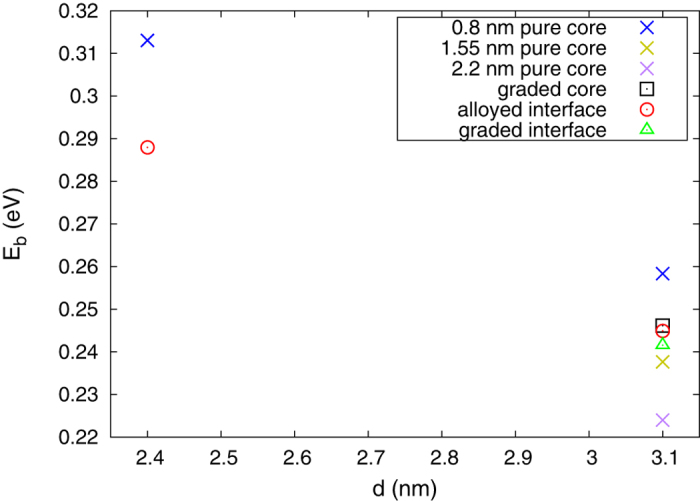
Electron-hole Coulomb interaction energies of CdSe/CdS core-shell NCs with different size, made using different structural models and core size.

However, in our calculations instead of using a nonlocal screening dielectric constant, *ε*(r_1_ − r_2_), we used a weighted average of the relative dielectric constants,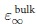 where for 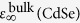 and 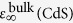 we used 6.2 and 5.4, respectively, which were taken from Ref. 47. The resulting calculated e-h interaction energies are plotted in Fig. 10.

From Fig. 10 one can see that the e-h Coulomb interaction energies get lower, as the size of the NCs increases, when comparing the NCs with same structural model. It is also noticeable that with enlarging core the e-h Coulomb interaction energy is decreasing. This can be attributed to the lowering of the confinement of the HOMO WF as the core gets bigger. Comparing the e-h *E*_*b*_ of the 3.1 nm NCs with alloyed interface and with pure core of the same size, 0.8 nm, it is evident that the structure with alloyed interface has a lower e-h *E*_*b*_. Keeping in mind that lower e-h *E*_*b*_ can increase the Auger recombination (AR) lifetimes^42^, which in turn will decrease the probability of AR, this lowering of the e-h *E*_*b*_ will contribute to the increase of the QE of the NCs with alloyed interface. Considering the previous discussion regarding the confinement of the HOMO WF and the Auger lifetimes, the decrease in the e-h *E*_*b*_ will further suppress the AR rates, indicating that the interfacial alloying can have significant influence on the AR in these NCs. In addition, both structures with graded core and graded interface have smaller e-h *E*_*b*_, compared to the NC with 0.8 nm pure core, indicating that having a graded core or graded interface can have a similar effect on the AR rates as the alloyed interface.

## Conclusions

In conclusion, we have carried out a thorough first-principle study of the effects of different interfaces and different core types in CdSe/CdS core-shell NCs on their properties. We also studied the changes in their properties with changes in the size of the NCs and the core/shell ratio. We show that the DOS and the absorption indices of the NCs with same diameter are very similar, regardless of the structural model. We observe the expected correlation between the size of the NCs and the band gaps—the band gaps decreases with increasing nanocrystal size. We observe a similar behaviour of the band gaps as in the experiments. A detailed inspection of spatial confinement of the HOMO and LUMO WFs within the core of the NCs, shows that the confinement of the HOMO WF depends sensitively on the core size, but not equally much on the interface. We argue that this change in the confinement of the HOMO WF, will have an influence on the QE of the NCs, as well as on the Auger lifetimes, as presented in the experiments. On the other hand the confinement of the LUMO WF is almost invariant in all of the studied structures. We demonstrate that the difference in the spatial distribution between the HOMO and LUMO causes difference in the radiative lifetimes, with the radiative lifetimes being similar for the structures with different structural model, but increase as the core gets smaller. Furthermore, we show that the electron-hole Coulomb interaction energies for the structures with same size, are higher in NCs with pure 0.8 nm core, compared to the other structural models, and are decreasing with increasing core size.

## Additional Information

**How to cite this article**: Kocevski, V. *et al.* First-principles study of the influence of different interfaces and core types on the properties of CdSe/CdS core-shell nanocrystals. *Sci. Rep.* **5**, 10865; doi: 10.1038/srep10865 (2015).

## Figures and Tables

**Table 1:** Number of Se, S and Cd atoms in pure CdSe NC, and CdSe/CdS core-shell NCs made using different structural models and different size.

**structural**	**NC diameter**							
**model**	**1.1 nm**		**2.4 nm**			**3.1 nm**		
	**Cd**	**Se**	**Cd**	**Se**	**S**	**Cd**	**Se**	**S**
pure core (2.2 nm)						321	152	160
purecore(1.55 nm)						321	68	244
purecore(0.8 nm)	13	16	141	16	36	321	16	296
graded core (2.2 nm)						321	61	251
alloyed interface^a^			141	68	84	321	68	244
graded interface^1^						321	73	239

^a^the interface including the CdSe core is 2.2 nm.
